# One‐Year Changes in Frailty Components After Curative Minimally Invasive Surgery for Colorectal Cancer in Older Patients

**DOI:** 10.1002/ags3.70269

**Published:** 2026-08-25

**Authors:** Hajime Ushigome, Takuya Suzuki, Shunsuke Hayakawa, Akira Kato, Yushi Yamakawa, Kenta Saito, Hiroyuki Sagawa, Ryo Ogawa, Hiroki Takahashi, Shuji Takiguchi

**Affiliations:** ^1^ Department of Gastroenterological Surgery Nagoya City University Graduate School of Medical Sciences Nagoya city Japan

**Keywords:** colorectal cancer, FRAIL Scale, frailty, Kihon checklist, minimally invasive surgery, older patients

## Abstract

**Background:**

Frailty predicts outcomes after colorectal cancer surgery, but postoperative changes remain unclear. We previously reported improvement in FRAIL Scale‐based frail/pre‐frail status 1 year after curative minimally invasive surgery (MIS). This study examined the components underlying that improvement using the FRAIL Scale and Kihon Checklist (KCL).

**Methods:**

This secondary analysis included patients aged ≥ 70 years who underwent curative MIS for colorectal cancer and had preoperative and 1‐year postoperative frailty assessments. Changes in FRAIL Scale and KCL indicators were evaluated using paired analyses.

**Results:**

Among 239 patients assessed preoperatively, 141 were included in the paired analysis. Among FRAIL Scale items, only the weight‐loss criterion improved significantly (35.8% to 9.5%, *p* < 0.001). In the KCL, oral function risk improved, whereas physical function risk tended to worsen; houseboundness, cognitive decline, and depressive mood were unchanged. Patients whose FRAIL Scale weight‐loss criterion resolved showed a modest 1.2‐kg increase (*p* = 0.038), whereas mean body weight remained unchanged overall. In sensitivity analyses, the improvement in oral function risk persisted after excluding patients with preoperative bowel obstruction (*p* = 0.014). After excluding patients receiving oxaliplatin‐containing adjuvant chemotherapy, physical function risk still tended to increase, and fear of falling remained significantly increased (*p* = 0.011).

**Conclusions:**

FRAIL Scale improvement was driven mainly by resolution of the weight‐loss criterion, with modest weight gain and no clear overall body‐weight recovery. KCL changes varied across domains and items, with improvement in some aspects but persistence or worsening in others, supporting a multidimensional interpretation of postoperative recovery in older patients.

## Abstract 2

Older patients with colorectal cancer showed heterogeneous frailty‐related changes 1 year after curative minimally invasive surgery. FRAIL Scale improvement was driven mainly by resolution of the weight‐loss criterion despite unchanged overall body weight, whereas KCL oral function improved but some physical function‐related findings worsened. These findings support multidimensional postoperative assessment using multiple instruments.

## 1 Introduction

Colorectal cancer (CRC) is one of the most common gastrointestinal malignancies in both Asian and Western countries. In recent years, the number of older patients with CRC has increased in parallel with global population aging, and the importance of CRC management in older adults has become increasingly evident [1, 2].

Surgical resection remains the cornerstone of curative treatment, even in older patients with CRC. However, older patients often have physical, psychological, and social vulnerabilities that cannot be fully explained by chronological age or comorbidities alone; this condition is generally referred to as frailty. Frailty has been reported to be associated with postoperative complications, long‐term outcomes, functional recovery, and quality of life, and is therefore an important factor when considering treatment decision‐making and postoperative survivorship in older patients with CRC [3, 4, 5, 6].

Minimally invasive surgery (MIS) is currently one of the standard surgical approaches for CRC [7, 8]. Although the usefulness of MIS in older patients has increasingly been reported, evidence regarding the clinical significance of MIS in frail patients remains limited [9, 10]. In addition, how frailty status changes over time after MIS and what clinical meaning such changes have remain to be fully clarified.

In our previous study [11], we investigated changes in frailty status using the FRAIL Scale and Kihon Checklist (KCL) in patients aged 70 years or older with CRC who remained recurrence‐free for 1 year after curative MIS. We found that frail/pre‐frail status based on the FRAIL Scale significantly improved 1 year after surgery. This improvement was accompanied by increases in hemoglobin level, Prognostic Nutritional Index, and Psoas Muscle Index, suggesting that recovery from cancer‐related wasting and improvement in nutritional status after tumor resection may have contributed to the observed improvement in FRAIL Scale‐based frailty status.

However, our previous study mainly evaluated overall changes in frailty classification based on the FRAIL Scale and KCL, and did not sufficiently examine item‐level or domain‐level changes within each assessment tool. The FRAIL Scale is a simple and internationally recognized screening tool that primarily assesses physical frailty [12, 13]. In contrast, the KCL is a 25‐item self‐administered questionnaire developed by the Japanese Ministry of Health, Labour and Welfare to identify functional decline and the future risk of care dependency among community‐dwelling older adults. It assesses multiple domains, including physical function, nutritional status, oral function, houseboundness, cognitive function, and depressive mood [12, 14, 15].

A systematic review by Sewo Sampaio et al. [16] explained that, although frailty was traditionally conceptualized primarily in terms of physical functional decline, the concept has evolved and is now understood as a multidimensional construct encompassing cognitive, psychological, social, and nutritional aspects in addition to the physical dimension. The review also characterized the KCL as a reliable instrument for assessing general frailty and its individual aspects in older adults and indicated its applicability to cross‐cultural studies. Accordingly, the KCL may be useful for evaluating a broad range of frailty‐related vulnerabilities beyond physical frailty alone.

Combining item‐level analysis of the FRAIL Scale with domain‐ and item‐level analyses of the KCL may therefore allow a more detailed and multidimensional assessment of postoperative changes that cannot be captured by overall frailty classification alone. The aim of this study was to further characterize changes in frailty‐related indicators during the first year after curative MIS in older patients with colorectal cancer through item‐level analysis of the FRAIL Scale and domain‐ and item‐level analyses of the KCL.

## 2 Materials and Methods

### 2.1 Study Design

This study was a secondary analysis of a prospective observational cohort conducted at Nagoya City University Hospital. Frailty assessments using standardized questionnaires were performed preoperatively and 1 year postoperatively in patients scheduled to undergo resection for primary colorectal cancer (CRC). Questionnaire data were collected prospectively, and the present study retrospectively analyzed patients who had available frailty assessments at both the preoperative and 1‐year postoperative time points.

Overall changes in frailty classification, short‐term surgical outcomes, and long‐term outcomes in this cohort have been reported previously. In the present study, to further characterize the postoperative changes in frailty observed in our previous report, we focused on changes in individual items and domains of the two frailty assessment tools.

### 2.2 Frailty Assessment

Frailty was assessed using the FRAIL Scale and the Kihon Checklist (KCL). The FRAIL Scale is a simple screening tool for physical frailty and consists of five items: fatigue, resistance, ambulation, illnesses, and weight loss. Each item is scored as 0 or 1 point, with a total score ranging from 0 to 5. Based on the total score, patients were classified as non‐frail (0 points), pre‐frail (1–2 points), or frail (≥ 3 points) [12, 13] (Table S1). In addition to overall FRAIL Scale classification, changes in each of the five FRAIL Scale items from the preoperative assessment to 1 year postoperatively were analyzed.

The KCL is a 25‐item self‐administered questionnaire and is a comprehensive frailty assessment tool that evaluates multiple domains, including physical function, nutritional status, oral function, houseboundness, cognitive function, and depressive mood. The total score ranges from 0 to 25, and patients were classified as non‐frail (0–3 points), pre‐frail (4–7 points), or frail (≥ 8 points). Domain‐specific risk was defined as follows: physical function, ≥ 3 of 5 items; nutritional status, 2 of 2 items; oral function, ≥ 2 of 3 items; houseboundness, ≥ 1 of 2 items; cognitive function, ≥ 1 of 3 items; and depressive mood, ≥ 2 of 5 items [12, 15] (Table S2).

For paired analyses of overall frailty classifications and KCL domain‐specific risks, only patients whose classifications could be determined at both time points based on the available responses were included. Individual‐item analyses included only patients with responses available at both time points.

### 2.3 Patient Population

Between November 2019 and August 2023, 685 patients underwent resection for primary CRC at Nagoya City University Hospital and were considered eligible for the original cohort. Patients were excluded if they were younger than 70 years, had stage IV disease, underwent open surgery, had another malignancy under treatment, underwent R1 or R2 resection, or had missing preoperative questionnaire data because of emergency surgery, patient refusal, or other reasons. Consequently, 239 patients with available preoperative frailty assessment were included in our previous analysis.

In the present study, among these 239 patients, we excluded those who were transferred to another hospital within 1 year after surgery, developed recurrence, developed another malignancy, died, were lost to follow‐up, refused to complete the questionnaire, or had missing 1‐year postoperative questionnaire data. Finally, 141 patients who underwent curative minimally invasive surgery, remained recurrence‐free at 1 year postoperatively, and had frailty assessments available both preoperatively and 1 year postoperatively were included in the present analysis. The patient selection flow is shown in Figure S1.

Demographic, clinical, tumor‐related, surgical, and treatment‐related variables were collected, including age, sex, body mass index, body weight, American Society of Anesthesiologists physical status [17], Charlson Comorbidity Index [18], tumor location, pathological stage, preoperative bowel obstruction, preoperative treatment, operative factors, postoperative adjuvant chemotherapy and regimen. Frailty status, nutritional status, and skeletal muscle mass were assessed using the FRAIL Scale, Kihon Checklist, Prognostic Nutritional Index (PNI) [19], and Psoas Muscle Index [20, 21], respectively.

### 2.4 Outcomes

This study evaluated changes in frailty classification based on the FRAIL Scale and KCL from the preoperative assessment to 1 year postoperatively. To further characterize changes in frailty classification, we also analyzed changes from preoperative assessment to 1 year postoperatively in KCL domain‐specific risks, selected KCL items, and individual FRAIL Scale items.

### 2.5 Statistical Analysis

Continuous variables are presented as medians and ranges, except for body weight, which is presented as means and standard deviations, and categorical variables are presented as numbers and percentages. The Wilcoxon rank‐sum test was used for continuous variables, and the chi‐square test was used for categorical variables. The Wilcoxon signed‐rank test was used to compare paired continuous variables between the preoperative and 1‐year postoperative assessments. The McNemar test was used to compare paired categorical variables. Individual FRAIL Scale items, KCL domain‐specific risks, and individual KCL items were treated as binary variables, and changes from preoperative assessment to 1 year postoperatively were evaluated using the McNemar test. Body weight was analyzed using the Wilcoxon signed‐rank test. Sensitivity analyses were performed after excluding patients who received postoperative adjuvant chemotherapy, patients with preoperative bowel obstruction, or patients who received oxaliplatin‐containing adjuvant chemotherapy, as appropriate for the finding being examined. A *p* value of < 0.05 was considered statistically significant. All statistical analyses were performed using JMP software, version 19.0.4 (SAS Institute, Cary, NC, USA).

## 3 Results

### 3.1 Patient Characteristics

The demographic, clinicopathological, and treatment characteristics of the 141 patients included in the paired longitudinal analysis are summarized in Table 1. The median age was 77 years, and 79 patients (56.0%) were men. Preoperative bowel obstruction was present in 22 patients (15.6%), and 26 patients (18.4%) received postoperative adjuvant chemotherapy, including 12 patients (8.5%) who received an oxaliplatin‐containing regimen. All patients underwent minimally invasive surgery, with laparoscopic and robotic approaches used in 68 (48.2%) and 73 (51.8%) patients, respectively. Baseline characteristics were additionally compared between the 141 patients included in the paired analysis and the 75 patients who were transferred to another hospital within 1 year after surgery (*n* = 15) or lacked 1‐year questionnaire data because of loss to follow‐up or missing responses (*n* = 60) (Table S3). The latter group had a lower proportion of patients with performance status 0–1 and a lower Prognostic Nutritional Index than the paired‐analysis cohort. No significant differences were observed in the preoperative FRAIL Scale score or category or in the KCL total score or category.

**TABLE 1:** Demographic, clinicopathological, surgical, and treatment‐related characteristics of patients included in the paired longitudinal analysis.

Variables, *n* (%) or median (range)	Paired longitudinal cohort (*n* = 141)
Demographic and baseline clinical characteristics	
Age (years)	77 (70–93)
Male/female	79:62
BMI (kg/m^2^)	21.7 (14.2–35.4)
Performance status	
0–1	135 (95.7)
2–4	6 (4.3)
ASA‐PS	
1–2	113 (80.1)
3–4	28 (19.9)
Charlson Comorbidity Index	
0–2	120 (85.1)
3–4	20 (14.2)
≥ 5	1 (0.7)
Prognostic Nutritional Index	48 (32.5–67.5)
Psoas Muscle Index	4.62 (1.8–10.9)
Low PMI	
Yes	82 (60.3)
No	54 (39.7)
Preoperative FRAIL Scale score	1 (0–5)
Preoperative KCL total score	5 (0–18)
Tumor and treatment characteristics	
Tumor location	
Colon	82 (58.2)
Rectum	59 (41.8)
Preoperative bowel obstruction	
Yes	22 (15.6)
No	119 (84.4)
Preoperative treatment	
None	138 (97.9)
Chemotherapy	1 (0.7)
Chemoradiotherapy	2 (1.4)
Pathological stage	
0	3 (2.1)
I	45 (31.9)
II	58 (41.3)
III	35 (24.8)
Adjuvant chemotherapy	
None	115 (81.6)
Fluoropyrimidine alone	14 (9.9)
Oxaliplatin‐containing regimen	12 (8.5)
Operative characteristics	
Surgical approach	
Laparoscopic	68 (48.2)
Robotic	73 (51.8)
Operation time (min)	240 (117–750)
Blood loss (mL)	30 (0–988)
Stoma creation	
Yes	32 (22.7)
No	109 (77.3)
Postoperative outcomes	
Postoperative complications (CD ≧ III)	4 (2.8)
Postoperative hospital stay (days)	10 (7–105)

Abbreviations: ASA‐PS, American Society of Anesthesiologists physical status classification system; BMI, body mass index; CD, Clavien–Dindo; PMI, psoas muscle index.

### 3.2 Changes in Frailty Classification Based on the FRAIL Scale and KCL

**FIGURE 1:**
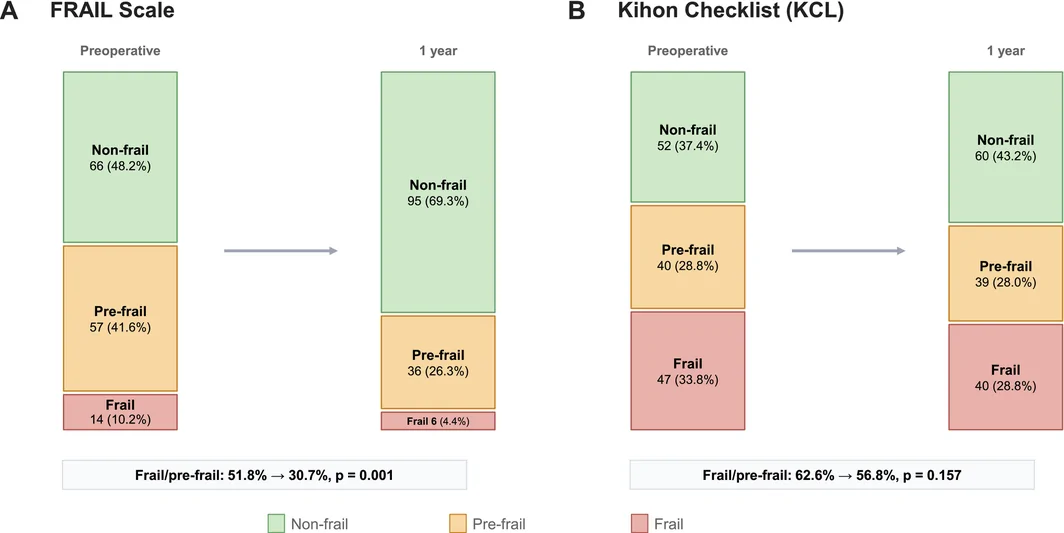
Changes in frailty category distributions before and 1 year after minimally invasive surgery. Frailty status was determined when the available responses permitted definitive classification. Paired analyses included patients classifiable at both the preoperative and one‐year assessments.

From the preoperative assessment to 1 year postoperatively, the proportion of patients classified as frail/pre‐frail based on the FRAIL Scale significantly decreased from 71 of 137 patients (51.8%) to 42 of 137 patients (30.7%) (*p* = 0.001). In contrast, the proportion of patients classified as frail/pre‐frail based on the KCL decreased from 87 of 139 patients (62.6%) to 79 of 139 patients (56.8%), but this change was not statistically significant (*p* = 0.157) (Figure 1).

### 3.3 Item‐Level Analysis of the FRAIL Scale

Preoperative‐to‐postoperative changes in individual FRAIL Scale items are shown in Table 2. Fatigue, resistance, ambulation, and illnesses all decreased at 1 year postoperatively, but none of these changes reached statistical significance. The number of patients meeting the illnesses criterion tended to decrease from 15 of 137 patients (10.9%) to 8 of 137 patients (5.8%), although the difference was not statistically significant (*p* = 0.071). In contrast, the number of patients meeting the weight loss criterion, defined as ≥ 5% weight loss, significantly decreased from 49 of 137 patients (35.8%) to 13 of 137 patients (9.5%) (*p* < 0.001).

**TABLE 2:** Changes in individual FRAIL Scale items between the preoperative and 1‐year postoperative assessments.

FRAIL Scale item	Paired *n*	Preoperative positive response, *n* (%)	1‐year postoperative positive response, *n* (%)	*p*
Do you feel tired most of the time?	137	28 (20.4)	20 (14.6)	0.144
Difficulty climbing one flight of stairs without rest	137	19 (13.9)	15 (10.9)	0.393
Difficulty walking one block unaided	137	15 (10.9)	12 (8.8)	0.466
Do you have five or more illnesses?	137	15 (10.9)	8 (5.8)	0.071
Have you lost more than 5% of your weight in the last year?	137	49 (35.8)	13 (9.5)	< 0.001

*Note:* Values indicate frailty‐positive responses. Paired *n* represents the number of patients with available responses at both assessments.

### 3.4 Changes in KCL Domain‐Specific Risks

Preoperative‐to‐postoperative changes in KCL domain‐specific risks are shown in Table 3. The number of patients with physical function risk tended to increase from 26 of 139 patients (18.7%) preoperatively to 35 of 139 patients (25.2%) at 1 year postoperatively, although the difference did not reach statistical significance (*p* = 0.072). Oral function risk significantly decreased from 31 of 138 patients (22.5%) to 19 of 138 patients (13.8%) (*p* = 0.018). There were no significant preoperative‐to‐postoperative changes in nutritional status risk, houseboundness risk, cognitive function risk, or depressive mood risk.

**TABLE 3:** Changes in Kihon Checklist domain‐specific risks and selected physical and oral function items between the preoperative and 1‐year postoperative assessments.

KCL domain	Paired *n*	Preoperative, *n* (%)	At 1‐year, *n* (%)	*p*
Domain‐specific risks				
Physical function	139	26 (18.7)	35 (25.2)	0.072
Nutritional status	139	6 (4.3)	4 (2.8)	0.527
Oral function	138	31 (22.5)	19 (13.8)	0.018
Houseboundness	140	13 (9.3)	15 (10.7)	0.654
Cognitive function	138	49 (35.5)	45 (32.6)	0.553
Depressive mood	139	51 (36.7)	46 (33.1)	0.317
Selected individual items				
Physical function				
Difficulty climbing stairs without using a handrail or wall for support	140	45 (32.1)	49 (35.0)	0.414
Difficulty standing up from a chair without any aids	140	25 (17.9)	34 (24.3)	0.038
Difficulty walking continuously for 15 min	140	21 (15.0)	20 (14.3)	0.841
Fall within the past year	139	27 (19.4)	25 (17.9)	0.745
Fear of falling while walking	139	51 (36.7)	68 (48.9)	0.007
Oral function				
Difficulty eating hard foods compared with 6 months earlier	138	33 (23.9)	24 (17.4)	0.072
Choking on tea or soup	140	27 (19.3)	27 (19.3)	1.000
Dry mouth	140	35 (25.0)	30 (21.4)	0.398

*Note:* Domain‐specific risk was classified when available responses allowed definitive determination. Paired analyses included patients classifiable at both assessments; therefore, sample sizes varied by domain and item.

### 3.5 Item‐Level Analysis of the KCL

Because domain‐level analyses suggested notable changes in physical function and oral function, related KCL items were further analyzed, as shown in Table 3. Among physical function items, the number of patients who had difficulty standing up from a chair without support significantly increased from 25 of 140 patients (17.9%) to 34 of 140 patients (24.3%) (*p* = 0.038). The number of patients who reported fear of falling while walking also significantly increased from 51 of 139 patients (36.7%) to 68 of 139 patients (48.9%) (*p* = 0.007). In contrast, there were no significant changes in the proportion of patients who had difficulty walking continuously for 15 min or who had fallen within the past year.

Among oral function items, the number of patients who reported difficulty eating hard foods compared with 6 months earlier tended to decrease from 33 of 138 patients (23.9%) to 24 of 138 patients (17.4%), although the difference was not statistically significant (*p* = 0.072). There were no significant changes in the proportions of patients who choked on tea or soup or who reported dry mouth.

### 3.6 Additional Analyses of Frailty‐Related Findings Showing Notable Changes

Additional analyses were performed to aid the clinical interpretation of FRAIL Scale and KCL findings that showed significant or suggestive changes in the primary analyses (Table 4). Specifically, actual changes in body weight were examined in relation to the FRAIL Scale weight‐loss item and the potential influence of postoperative adjuvant chemotherapy. For the KCL findings, the potential influence of oxaliplatin‐containing postoperative adjuvant chemotherapy was considered in the physical function‐related analyses, whereas the potential influence of preoperative bowel obstruction was considered in the oral function‐related analyses.

**TABLE 4:** Sensitivity analyses of key frailty‐related findings after accounting for potential confounding factors.

(A) Body‐weight changes related to the FRAIL Scale weight‐loss criterion and postoperative adjuvant chemotherapy					
Analysis group	*n*	Preoperative BW, kg	BW at 1 year, kg	Change in BW, kg	*p*
Overall cohort	136	56.4 ± 10.3	56.7 ± 10.3	0.3 ± 4.5	0.955
Excluding postoperative adjuvant chemotherapy	110	56.3 ± 10.3	56.6 ± 10.5	0.3 ± 4.2	0.741
Resolution of the FRAIL Scale weight‐loss criterion	43	56.7 ± 11.2	57.9 ± 10.7	1.2 ± 3.5	0.038
Excluding postoperative adjuvant chemotherapy	35	55.1 ± 11.3	56.3 ± 10.5	1.1 ± 3.4	0.076

(B) Changes in physical‐function indicators after excluding patients who received oxaliplatin‐containing adjuvant chemotherapy				
Outcome	*n*	Preoperative, *n* (%)	At 1‐year, *n* (%)	*p*
Domain‐specific risks				
Physical function	127	24 (18.9)	33 (26.0)	0.072
Selected individual items				
Physical function				
Difficulty climbing stairs without using a handrail or wall for support	127	43 (33.9)	46 (36.2)	0.532
Difficulty standing up from a chair without any aids	127	24 (18.9)	31 (24.4)	0.089
Difficulty walking continuously for 15 min	127	19 (14.9)	19 (14.9)	1.000
Fall within the past year	127	26 (20.5)	22 (17.3)	0.505
Fear of falling while walking	127	48 (37.8)	63 (49.6)	0.011

(C) Changes in KCL oral‐function indicators after excluding patients with preoperative bowel obstruction				
Outcome	*n*	Preoperative, *n* (%)	At 1‐year, *n* (%)	*p*
Domain‐specific risks				
Oral function	116	28 (24.1)	16 (13.8)	0.014
Selected individual items				
Oral function				
Difficulty eating hard foods compared with 6 months earlier	116	30 (25.9)	22 (19.0)	0.081
Choking on tea or soup	118	23 (19.5)	22 (18.6)	0.847
Dry mouth	118	33 (28.0)	25 (21.2)	0.157

*Note:* Body‐weight values and changes are presented as mean ± standard deviation. The FRAIL Scale weight‐loss item was defined as an unintentional weight loss of ≥ 5% during the preceding year. Body‐weight change was calculated as body weight at 1 year minus preoperative body weight. In Panels B and C, sample sizes varied by outcome because analyses included only patients classifiable at both assessments after the specified exclusions.

Among the 136 patients with body‐weight data available at both assessments, mean body weight did not significantly change from 56.4 ± 10.3 kg preoperatively to 56.7 ± 10.3 kg at 1 year postoperatively (mean change, 0.3 ± 4.5 kg; *p* = 0.955). The result was similar after excluding patients who received postoperative adjuvant chemotherapy (56.3 ± 10.3 kg vs. 56.6 ± 10.5 kg; mean change, 0.3 ± 4.2 kg; *p* = 0.741). In contrast, among the 43 patients whose FRAIL Scale weight‐loss criterion changed from positive preoperatively to negative at 1 year postoperatively, body weight increased from 56.7 ± 11.2 kg to 57.9 ± 10.7 kg (mean change, 1.2 ± 3.5 kg; *p* = 0.038). After excluding patients who received postoperative adjuvant chemotherapy, the increase remained similar in magnitude but was no longer statistically significant (55.1 ± 11.3 kg vs. 56.3 ± 10.5 kg; mean change, 1.1 ± 3.4 kg; *p* = 0.076).

After excluding the 12 patients who received oxaliplatin‐containing postoperative adjuvant chemotherapy, physical function risk still tended to increase from 24 of 127 patients (18.9%) to 33 of 127 patients (26.0%), although the difference was not statistically significant (*p* = 0.072). The increase in difficulty standing up from a chair was no longer statistically significant, whereas fear of falling while walking remained significantly increased.

After excluding 22 patients with preoperative bowel obstruction, oral function risk remained significantly decreased from 28 of 116 patients (24.1%) to 16 of 116 patients (13.8%) (*p* = 0.014). Difficulty eating hard foods also showed a nonsignificant decrease.

## 4 Discussion

In this study, we examined changes in frailty‐related indicators during the first year after curative minimally invasive surgery (MIS) for colorectal cancer in older patients through item‐level analysis of the FRAIL Scale and domain‐ and item‐level analyses of the KCL. Item‐level analysis of the FRAIL Scale indicated that the improvement in frail/pre‐frail status was primarily explained by resolution of the weight‐loss criterion. In the KCL domain‐level analysis, oral function risk significantly improved, whereas no clear changes were observed in houseboundness, cognitive function, or depressive mood. In contrast, physical function risk tended to increase, and item‐level analysis showed significant increases in difficulty rising from a chair and fear of falling. These findings indicate that postoperative changes differed according to the assessment tool and domain evaluated.

In light of the present findings, the improvement in FRAIL Scale‐based frail/pre‐frail status observed in our previous study [11] appears to have reflected primarily the resolution of the weight‐loss criterion rather than a broad recovery of physical function. However, additional analyses showed that body weight remained largely unchanged in the overall cohort from the preoperative assessment to 1 year after surgery. Even among patients in whom the weight‐loss criterion became negative, the mean increase in body weight was limited to approximately 1 kg. Therefore, this finding should not be interpreted as evidence of clear overall weight recovery.

Rather than indicating substantial weight gain, resolution of the weight‐loss criterion may have reflected cessation or attenuation of preoperative weight loss. This interpretation is consistent with established definitions and pathophysiological concepts of cancer cachexia [22, 23], as well as the concept of cancer‐related frailty [4]. In patients with colorectal cancer, cancer‐related inflammation, reduced dietary intake, metabolic abnormalities, and anemia may contribute to vulnerability accompanied by weight loss and skeletal muscle loss. Alleviation of some of these cancer‐related factors after curative resection may have contributed to the observed change. The direction of body‐weight change was similar after excluding patients who received postoperative adjuvant chemotherapy. Nevertheless, the present study did not directly evaluate the mechanisms underlying cancer‐related wasting, and the actual increase in body weight was limited. Therefore, this interpretation remains hypothetical, and the underlying mechanisms require further investigation.

In the KCL‐based assessment, frail/pre‐frail status did not significantly improve 1 year after surgery; however, among the KCL domains, only oral function risk significantly improved. In the item‐level analysis of oral function, the proportion of patients who reported difficulty eating hard foods compared with 6 months earlier tended to decrease. These improvements were also observed in sensitivity analyses excluding patients with preoperative bowel obstruction. In cancer cachexia, in addition to weight loss and muscle loss, nutrition‐impact symptoms such as anorexia, taste alterations, dysphagia, and dry mouth are considered clinically important [23]. Therefore, improvement in KCL oral function risk, together with improvement in the weight loss item of the FRAIL Scale, may reflect alleviation of symptoms affecting nutritional intake after curative resection. However, because dietary intake and objective oral function were not directly assessed in this study, the underlying mechanisms require further investigation, as does the interpretation of the FRAIL Scale weight‐loss criterion.

In contrast, KCL‐defined physical function risk did not improve but tended to increase. At the item level, no clear changes were observed in difficulty walking continuously for 15 min or history of falls, whereas difficulty rising from a chair and fear of falling significantly increased. Notably, the increase in fear of falling persisted in the sensitivity analysis excluding patients who received postoperative adjuvant chemotherapy containing oxaliplatin. These findings suggest that even when the weight‐loss criterion resolves and oral function risk improves, physical vulnerability in daily life, as captured by the KCL, may not improve in parallel.

The differences in physical function‐related findings between the FRAIL Scale and the KCL observed in this study are not necessarily contradictory. The resistance and ambulation items of the FRAIL Scale assess basic mobility, such as the ability to climb stairs and walk. In contrast, the KCL more broadly assesses physical function and related aspects of daily life, including difficulty rising from a chair, history of falls, and fear of falling. Previous studies have shown that chair‐rise ability reflects not only lower‐extremity muscle strength but also balance, sensorimotor function, and mobility [24]. Fear of falling also reflects subjective and psychological aspects related to physical function [25, 26]. Thus, the FRAIL Scale findings suggest that basic mobility was generally maintained, whereas the KCL may have captured functional and subjective changes in daily life. Interpreting the findings from the two instruments as complementary may provide a more comprehensive understanding of postoperative recovery.

These findings highlight the need to monitor not only recurrence but also frailty and functional recovery during postoperative follow‐up in older patients with colorectal cancer. Even in the era of minimally invasive surgery, physical limitations may persist in some older patients [27], potentially owing to incomplete recovery from postoperative deconditioning as well as age‐related declines in muscle strength, balance, and mobility [28]. Multidimensional frailty assessment may therefore help identify patients who could benefit from rehabilitation, exercise, or fall‐prevention guidance [29]. Such interventions may also improve psychosocial outcomes, including depression, anxiety, and quality of life [30].

This study has several limitations. First, this was a subanalysis of a single‐center prospective observational cohort, and the sample size was limited. Second, the longitudinal analyses were limited to the 141 patients with both preoperative and 1‐year postoperative assessments. Of the 239 eligible patients, 98 were not included in the paired analyses, including 75 who did not undergo the 1‐year assessment because of transfer to another hospital (*n* = 15), loss to follow‐up, or missing questionnaire data (*n* = 60). As shown in Table S3, patients with paired assessments had better PS and a higher PNI than those without a 1‐year assessment, suggesting that the analyzed cohort may have been biased toward patients with a more favorable baseline condition. Therefore, the findings reflect changes among patients who remained recurrence‐free and were assessed at both time points, and may not be fully generalizable to those with poorer baseline status or an unfavorable postoperative course. Third, this study was based on questionnaire‐based assessments and did not include objective physical function measures such as grip strength, gait speed, muscle strength, or balance function. Peripheral neuropathy potentially associated with oxaliplatin‐containing adjuvant chemotherapy and objective oral or swallowing function were also not directly assessed; therefore, the mechanisms underlying the observed physical and oral function‐related changes could not be fully determined. Fourth, the item‐level analyses were exploratory, and the influence of multiple comparisons cannot be completely excluded.

Despite these limitations, studies examining longitudinal changes in frailty after surgery in older patients with colorectal cancer remain limited. In particular, few studies have evaluated item‐ and domain‐level changes using different assessment tools. The present study showed that postoperative frailty‐related changes differed according to the assessment tool and domain evaluated.

In conclusion, FRAIL Scale‐based frail/pre‐frail status improved 1 year after curative MIS in older patients with colorectal cancer, largely owing to resolution of the weight‐loss criterion. However, body weight remained unchanged in the overall cohort, and patients with resolution of this criterion showed a modest mean weight gain of approximately 1 kg. In contrast, KCL‐based assessment showed improvement in oral function risk but persistent or worsening physical function‐related vulnerability. These findings suggest that postoperative frailty‐related changes are heterogeneous and may differ according to the construct and assessment instrument evaluated. Assessment using instruments that capture different dimensions of frailty may provide a more comprehensive understanding of postoperative recovery.

## Author Contributions

**Hajime Ushigome:** conceptualization, methodology, writing – original draft, writing – review and editing, investigation, data curation, formal analysis, project administration. **Takuya Suzuki:** conceptualization, investigation, data curation, writing – review and editing, methodology, supervision, project administration. **Kenta Saito:** conceptualization, methodology. **Shuji Takiguchi:** conceptualization, supervision. **Hiroyuki Sagawa:** conceptualization, methodology. **Shunsuke Hayakawa:** conceptualization, methodology. **Yushi Yamakawa:** investigation, data curation, writing – review and editing. **Hiroki Takahashi:** conceptualization, supervision, investigation, data curation. **Ryo Ogawa:** conceptualization, supervision, methodology. **Akira Kato:** investigation, data curation.

## Funding

The authors have nothing to report.

## Ethics Statement

S.T. is an editorial board member of Annals of Gastroenterological Surgery. The study protocol was approved by the institutional review board (Nos. 60‐19‐0150 and 60‐19‐0064). Written informed consent was waived due to the retrospective nature of the study. For the prospective frailty assessment (IRB No. 60‐19‐0064), consent was obtained through an explanatory document and participant agreement via checkbox.

## Conflicts of Interest

The authors declare no conflicts of interest.

## Supporting information


**Figure S1:** Patient selection flow for the paired longitudinal frailty analysis.

**Table S1:** FRAIL Scale scoring criteria.

**Table S2:** Kihon Checklist scoring criteria.

**Table S3:** Comparison of clinical and perioperative characteristics between patients included in the paired analysis and those without a 1‐year assessment.

## Data Availability

The data that support the findings of this study are available on request from the corresponding author. The data are not publicly available due to privacy or ethical restrictions.
